# Taurine and Methylprednisolone Administration at Close Proximity to the Onset of Muscle Degeneration Is Ineffective at Attenuating Force Loss in the Hind-Limb of 28 Days *Mdx* Mice

**DOI:** 10.3390/sports6040109

**Published:** 2018-09-30

**Authors:** Robert G. Barker, Chris van der Poel, Deanna Horvath, Robyn M. Murphy

**Affiliations:** 1 Department of Biochemistry and Genetics, La Trobe Institute for Molecular Science, La Trobe University, Melbourne, VIC 3086, Australia; Robert.Barker@latrobe.edu.au; 2 Department of Physiology, Anatomy and Microbiology, La Trobe University, Melbourne, VIC 3086, Australia; C.VanDerPoel@latrobe.edu.au (C.v.d.P.); Deanna.Horvath@latrobe.edu.au (D.H.)

**Keywords:** DMD, *mdx* mouse, taurine, skeletal muscle, animal model, methylprednisolone

## Abstract

An increasing number of studies have shown supplementation with the amino acid taurine to have promise in ameliorating dystrophic symptoms in the *mdx* mouse model of Duchenne Muscular Dystrophy (DMD). Here we build on this limited body of work by investigating the efficacy of supplementing *mdx* mice with taurine postnatally at a time suggestive of when dystrophic symptoms would begin to manifest in humans, and when treatments would likely begin. *Mdx* mice were given either taurine (*mdx* tau), the steroid alpha methylprednisolone (PDN), or tau + PDN (*mdx* tau + PDN). Taurine (2.5% wt/vol) enriched drinking water was given from 14 days and PDN (1 mg/kg daily) from 18 days. Wild-type (WT, C57BL10/ScSn) mice were used as a control to *mdx* mice to represent healthy tissue. In the *mdx* mouse, peak damage occurs at 28 days, and in situ assessment of contractile characteristics showed that taurine, PDN, and the combined taurine + PDN treatment was ineffective at attenuating the force loss experienced by *mdx* mice. Given the benefits of taurine as well as methylprednisolone reported previously, when supplemented at close proximity to the onset of severity muscle degeneration these benefits are no longer apparent.

## 1. Introduction

Currently, steroids are the only effective treatment for Duchenne Muscular Dystrophy (DMD) [1,2]. While the method of action is not completely understood, it is widely accepted that their potent anti-inflammatory properties [1,3] are of great benefit to DMD patients, extending ambulation by several years [1,3,4,5] and improving life quality. There are currently no steadfast guidelines as to when steroid therapy should be implemented, with therapy typically beginning when the benefit outweighs the adverse side effects associated with the treatment (for a review, see Bushby, et al. [1]. Due to these side effects, there is a pressing need for safer alternatives.

Taurine is an amino acid found ubiquitously in all mammalian cells, identified as vital for healthy muscle function and development [6,7]. In the *mdx* mouse limb muscle, taurine levels have been found to be reduced both before and during the onset of severe dystropathology (21–28 days), before returning to endogenous levels as the pathology stabilises into adulthood (70 days) [8,9]. The availability of sufficient taurine both before and during these critical stages is therefore an area of interest for the potential attenuation of the dystrophic pathology shown in the *mdx* mouse.

In our previous work, prenatal supplementation of taurine was found to be effective at reducing the severity of dystrophic symptoms in peak damage 28 days *mdx* mice, increasing both muscle strength and the visible markers of muscle health [10]. However, it is currently unclear what effect taurine supplementation has if given post birth, in pre-weaned, juvenile *mdx* mice. Understanding this would provide a better insight into the effectiveness of taurine as a nutritional supplement after diagnosis in DMD patients.

While there is a limited body of work investigating taurine supplementation in the juvenile *mdx* mouse, a recent study found that 4% taurine supplementation in chow from 14 days of age was sufficient to greatly reduce myofibre necrosis and markers of inflammation in 22 days *mdx* mice [11]; however, no contractile characteristics or makers of strength were investigated in that study. Of note, hind-limb muscle deterioration, including atrophy and loss of function, in *mdx* mice begins at approximately 21 days following an increase in activity; thus, mice were investigated at the very beginning of the degenerative period [11], as opposed to peak damage at 28 days [12,13].

To better understand the potential of taurine as a substance of therapeutic benefit to DMD, the current study compares the effectiveness of beginning administration of taurine 14 days postnatally with the steroid methylprednisolone (PDN) on peak damage 28 days *mdx* mice. We further assess a combined treatment of tau + PDN in 28 days mice, with a dose similar to that previously reported by Cozzoli, et al. [14] but using the more physiologically relevant model of the non-exercised juvenile model, as opposed to adults that have been exercised to exacerbate the phenotype.

Taurine, PDN, and the combined taurine + PDN treatment was ineffective at attenuating the force loss experienced by *mdx* mice when supplemented at close proximity to the onset of severity muscle degeneration. This has important implications for the practical use of taurine as a substance of therapeutic benefit to DMD, as the same dosage of taurine given prenatally was found to be effective [10]. The findings suggest that the benefit of taurine potentially lies in its ability to reduce the initial onset of disease severity, as opposed to attenuating damage or downstream pathways once the pathology has started to progress.

## 2. Materials and Methods

### 2.1. Animals and Supplementation

All procedures in this study were approved by the La Trobe University Animal Ethics Committee (AEC 12–31, 13–48). Only male mice were used for experiments. A total of 26 *mdx* and 10 wild-type (WT, C57/BL10ScSn) were used. Experimental animals were bred at the La Trobe Animal Research and Teaching Facility (Victoria, Australia) using breeding pairs obtained from the Animal Resource Centre (Western Australia, Australia). The offspring of WT and *mdx* mice had access to standard rodent chow and water ad libitum, and were utilised for experimentation at 28 ± 1 days of age. The absence of dystrophin in *mdx* mice was confirmed by Western Blot, as previously described [10]. At 14 days, *mdx* taurine (tau) and *mdx* taurine + steroid (tau + PDN) mice were supplemented with continuous access to taurine (2.5% wt/vol) enriched drinking water, as this dosage of supplementation has been previously demonstrated to elevate skeletal muscle taurine content in *mdx* mice [10]. Taurine supplementation began at 14 days to allow time for taurine to be adequately ingested before experimentation. At 18 days, *mdx* PDN and *mdx* tau + PDN mice received a 1 mg/kg dose of alpha methylprednisolone (Methylpred, CAS: 2375-03-3) daily via intraperitoneal (I.P.) injection on alternate sides of the body until the day of experimentation at 28 days. This dosage has been identified previously as effective at increasing muscular strength in the *mdx* mouse [15,16]. As rapid weight gain was expected both from growth and as a result of PDN administration, I.P. injections allow a 1 mg/kg dosage to be administered accurately on subsequent days. I.P. injections of PDN were first administered from 18 days, as this was found to be the earliest pre-pups could be handled without risking abandonment. No treatments were given to WT mice.

### 2.2. Muscle Preparation for in Situ Contractile Protocol

Mice were anesthetised with an I.P. injection of 10 µL·g^−1^ Nembutal (Sodium Pentobarbitone) and kept unresponsive for the duration of the experiment with supplementary doses of Nembutal (10% initial volume). The mouse was weighed, placed on a 37 °C heated pad, and the right leg was skinned to the waist taking care not to damage the fascia. Throughout the experiment, warmed physiological saline (0.9%) was applied to exposed muscle tissue. The distal tendon of the tibialis anterior (TA) muscle was isolated and secured with both a top and bottom knot as close to the myotendinous junction as possible using 5.0 surgical thread (Johnson & Johnson, New Brunswick, NJ, USA). The distal tendon was then severed, and the TA dissected free from the surrounding tissue keeping nerve and blood flow intact at the tibia below the tibial plateau. The sciatic nerve was exposed by gently parting the midline of the biceps femoris muscle above the knee joint. The mouse was secured on the heated platform (37 °C) of an in situ contractile apparatus (809B in situ Mouse Apparatus, Aurora Scientific, Aurora, ON, Canada) with a pin behind the patellar tendon and a foot clamp. The distal end of the TA was tied firmly to a lever arm attached to an isometric force transducer. The sciatic nerve was stimulated by two field stimulating platinum electrodes coupled to an amplifier.

### 2.3. Contractile Protocol

The TA was contracted via square wave (0.2 ms) pulses at 10 V from the stimulator (701C stimulator, Aurora Scientific, Aurora, ON, Canada). Forces were converted to a digital signal and recorded by Dynamic muscle analysis 611A (DMA) (Aurora Scientific, Aurora, ON, Canada). Optimum muscle length (L_o_) was first determined by eliciting twitch contractions by incrementally adjusting muscle length with a micromanipulator until a repeatable maximum peak twitch force was obtained. Muscle length at L_o_ was measured with precision digital callipers from the beginning of the distal tendon to the insertion of the TA at the base of the knee. Subsequently, the TA was stimulated at 100 Hz tetanic contraction, followed by a 2 min rest interval, and then twitch contraction. Comparable twitch forces pre and post 100 Hz stimulation indicated that the knots were both secure and unlikely to slip during the remaining protocol. If a decrease in twitch force was observed, the muscle was incrementally tensioned and stimulated between 2 min rest intervals until peak twitch force (P_t_) was re-established. To establish the force frequency relationship, the TA was stimulated supramaximally (10 V) for 500 ms at 10, 20, 30, 40, 50, 80, 100, 150, and 200 Hz, with a 2 min rest interval in-between. Maximum isometric tetanic (P_o_) force was determined from the largest force produced during the force-frequency stimulation protocol. Maximum tetanic specific force (sP_o_) was determined as force per cross sectional area (CSA) as described by Lynch (DMD_M.2.2.005). Briefly, optimum fibre length (L_f_) was first determined by multiplying L_o_ by the predetermined TA length to fibre length ratio of 0.6 [17], and utilising the formula sP_o_ = P_o_ × (muscle mass/L_f_ × 1.06).

Following the force frequency protocol, the TA was allowed a 2 min recovery prior to commencement of the fatigue protocol. Fatigue was induced by stimulating the TA for 500 ms at 60 Hz every second for 180 s. At the completion of the fatigue protocol, recovery was assessed by stimulating the muscle for 500 ms at 60 Hz following 1, 2, and 3 min recovery periods.

Immediately following the contraction protocol, the mouse was removed from the apparatus and the contractile TA dissected, blotted clean on filter paper (Whatman No. 1), and weighed. The mouse was then killed by cardiac excision.

### 2.4. Statistics

All data are presented as mean ± standard deviation (SD) unless stated otherwise. Comparisons between WT and *mdx*; *mdx*, *mdx* tau, *mdx* PDN and *mdx* tau + PDN groups were performed using a one-way ANOVA of variance with individual stimulation frequencies in the force-frequency analysis, and individual time points in the fatigue and recovery analyses, with Sidak’s post-hoc analyses. All statistical analysis was performed using GraphPad Prism v 6. Significance was set at *p* < 0.05.

## 3. Results

### 3.1. Effect of Taurine Supplementation on Body Mass, Muscle Mass, and in Situ Contractile Characteristics of Maximum and Specific Force Production

**Figure 1:**
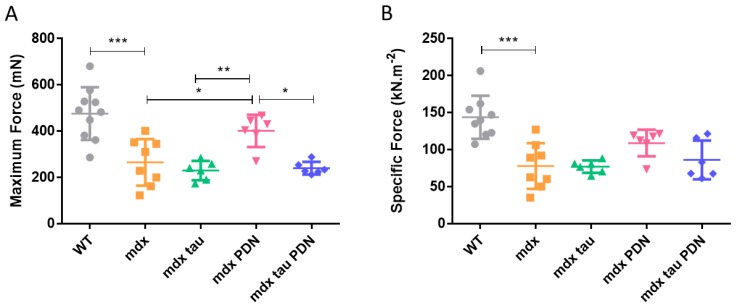
In situ force production in tibialis anterior muscles from 28 days WT, *mdx* and *mdx* tau, *mdx* PDN, and *mdx* tau + PDN treated mice. In situ maximum force (**A**) and specific force (**B**) Lines above specific bars indicate significant differences between groups (* *p* < 0.05, ** *p* < 0.01, *** *p* < 0.001). One-way ANOVA with Sidak’s post-hoc analyses. Data presented as means ± SD with n indicated by number of symbols.

Neither taurine, PDN nor the combined tau + PDN treatment had an effect on body or TA mass in *mdx* mice (Table 1). Figure 1A,B show the mean responses for in situ maximum and specific contractile force for all groups. Both maximum and specific forces in the 28 days *mdx* mice were ~50% weaker than the WT group (Figure 1A,B). Whilst maximum force production in *mdx* PDN mice was significantly greater than all other *mdx* groups, when normalised to the subtle differences in TA muscle mass (Table 1) there was no significant difference in muscle strength (Figure 1A,B). Table 1 also shows the mean values for in situ twitch and tetanic TA muscle characteristics. Despite lower absolute and specific forces, *mdx* mice had significantly greater optimum length (L_o_), time to peak tension (TTP), and half-relaxation time (½RT) than the WT group (Table 1). The P_t_ value of *mdx* PDN mice was significantly greater than all other *mdx* groups, with no change in any other contractile characteristics (Table 1).

### 3.2. Force Frequency Relationship

**Figure 2:**
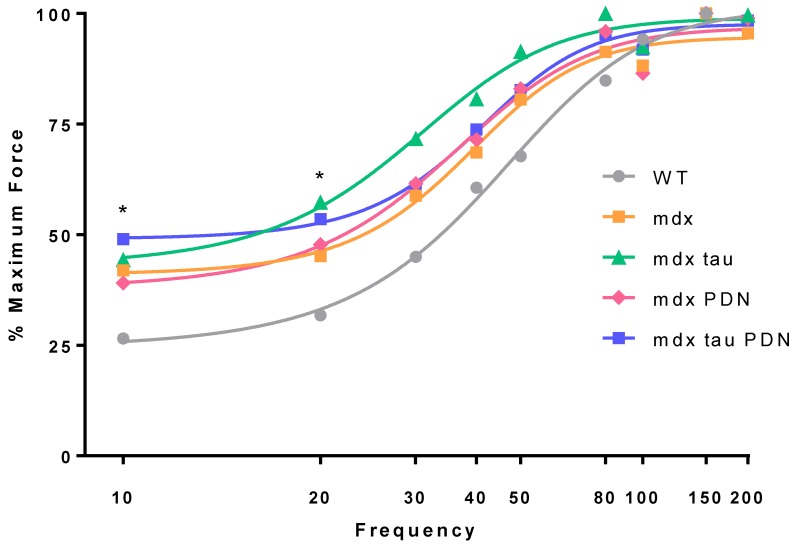
Force frequency relationship 28 days WT, *mdx* and *mdx* taurine (*mdx* tau), prednisolone (*mdx* PDN), taurine and prednisolone (*mdx* tau + PDN) treated mice. Data presented as mean percentage of maximum force output, error bars have been removed for clarity (WT n = 10, *mdx* n = 8, *mdx* tau n = 6, *mdx* PDN n = 6, *mdx* tau + PDN n = 6). One-way ANOVA with Sidak’s post-hoc analyses within relevant groups at given frequency. * *p* < 0.05, WT vs. 28 days *mdx*.

Neither tau, PDN, nor tau + PDN had an effect on the force frequency response in *mdx* mice (Figure 2). At 10 and 20 Hz, the TA muscle from *mdx* mice was more sensitive to the stimuli than WT animals, producing a significantly greater percentage of maximum force at these frequencies (Figure 2).

### 3.3. Fatigue Recovery

**Figure 3:**
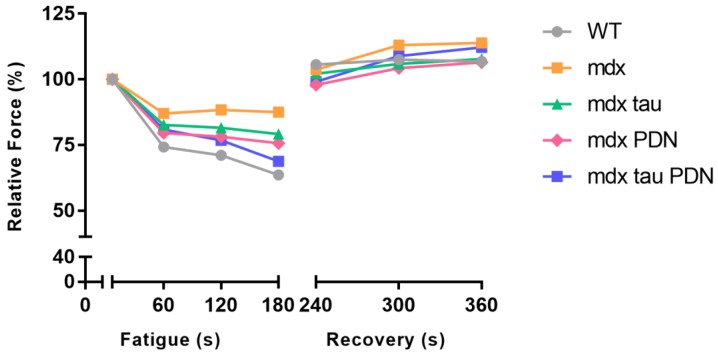
60 Hz fatigue and recovery from 28 days WT, *mdx*, *mdx* tau, *mdx* PDN and *mdx* tau + PDN treated mice. Data expressed as percentage of force relative to time zero, and presented as means, error bars have been removed for clarity (WT n = 8, *mdx* n = 8, *mdx* tau n = 6, *mdx* PDN n = 6, *mdx* tau + PDN n = 6). One-way ANOVA with Sidak’s post-hoc analyses within relevant groups.

In response to a low stimulation fatigue protocol there was no difference in the rate of fatigue or recovery between all *mdx* groups when expressed as % of initial force (Table 2, Figure 3). Post-tetanic potentiation was observed with subsequent recovery stimuli in all groups (Table 2, Figure 3).

## 4. Discussion

A growing number of studies have reported the postnatal administration of taurine to be effective at reducing the dystropathology of *mdx* mice; however, these have either been demonstrated in adult *mdx* mice (>6 weeks) [14,16,18,19,20], where the natural recovery of this model into adulthood may act to confound results, or in juvenile mice, where peak damage has yet to be reached but with no functional data reported [11].

In the present study, we identified that the continuous administration of taurine in 14 days old pre-weaned, juvenile *mdx* mice, elicited no improvement in in situ contractile strength or fatigue/recovery characteristics when assessed at 28 days of age (Figure 1 and Figure 2). While remaining side effect free, this finding is in contrast to the improvement in muscle strength that we have observed with the same supplementary dosage of taurine and contractile protocol with prenatal taurine supplementation [10], as well as when compared to the previous *mdx* mouse studies postnatally supplementing with taurine [14,16,18,19,20]. Importantly, in murine models, taurine metabolism and uptake are highly variable in different tissues [21], as well as with age, with plasma to tissue exchange rates found to be faster in adult as opposed to young mice (7 days) [22]. Therefore, the age of the mice used (peak naturally occurring pathology, 28 days), an in situ functional assessment, and the timing and duration of the postnatal supplementation are all important considerations when attempting to draw comparisons and conclusions from other studies supplementing with taurine.

Most relevant to the current study is the work by Terrill, Grounds and Arthur [11], who similarly began to administer taurine in *mdx* mice at 14 days (4% in chow), and found 8 days of taurine supplementation resulted in a 40% increase in hind limb muscle taurine content which was accompanied by 75% reduction myofibre necrosis, as well as a significant reduction in inflammatory markers [11]. It is unclear if this translated to an improvement in muscle function, as no contractile data were collected in this study. However, the striking visual reduction in muscle damage reported certainly suggests a functional improvement would be likely [11]. Considering the severity of muscle degeneration in dystrophy is relative to contractile activity, and the onset of activity post-weaning (~21 days) is believed to be one of the root causes of the severe onset of muscle degeneration seen in *mdx* mice [23], the improvement seen at 22 days [11] may reflect a lack of damage, or repeated bouts of damage yet to take place. Certainly, in the present study where mice were at 28 days of age and had undergone an additional 6 days of muscle damage, there was no improvement in contractile force production with taurine supplementation.

In a similar study, mice were assessed beyond the peak damage period 21–28 days of age [18]. *Mdx* mice were supplemented with taurine in drinking water (2%) from 18 days of age with functional assessment taking place at 42 days of age (adult mice) following 24 days of supplementation. In that study, functional improvement was observed both in vivo when assessed by grip strength, and ex vivo in an isolated extensor digitorum longus (EDL) preparation. It is unclear if the natural regeneration of the *mdx* mouse may have facilitated the improvement in strength seen in that study [18]. A reduction in tissue taurine content is observed in *mdx* mice during the damage period of ~21–28 days of age, but similar tissue taurine content is observed between *mdx* and WT mice both before and after this damage period (e.g. 18, 22, 36 and 42 days) [11,18,24]. The results from the present study and those described above, determine that, ultimately, more research is required to assess skeletal muscle function, as well as markers of pathological progression with taurine throughout the life history of the *mdx* mouse, with particular emphasis from juvenile (~2 weeks) to adults (>6 weeks) mice in order to assess where/when taurine is the most efficacious.

The *mdx* mice receiving the PDN treatment from 18 days of age produced significantly greater peak twitch and maximum force than all other *mdx* groups, including the *mdx* tau + PDN group (Table 1, Figure 1). However, while the TA mass in *mdx* PDN mice was not statistically greater than that of the other groups (Table 1), when taking it into account and force expressed relative to muscle mass, all *mdx* groups were comparable in strength (Figure 1B). No other differences in contractile characteristics were identified between *mdx* groups (Table 1). Why the combined tau + PDN treatment was seemingly incompatible because of the reduced beneficial effect on maximum force production than the PDN treatment alone is unclear. At 28–35 days, *mdx* mice exercised (to induce a more severe phenotype) and given a similar dosage of methyl-prednisolone (1 mg·kg^−1^, I.P.), taurine (1 g/kg/day) or a combination of both for 4–8 weeks, in vivo forelimb grip strength was increased [14]. In that study, the mechanical threshold of *mdx* mice was shifted to more positive potentials, and the combined tau + PDN treatment was more efficacious than either treatment individually. This finding was not replicated here with a similar dosage scheme; however, the differences in mouse age and functional assessment make direct comparisons unrealistic.

PDN has been identified to increase muscle function and prolong ambulation in DMD patients on numerous occasions, albeit with side effects [5,25,26]. It was hypothesised that due to the anabolic as well as potent anti-inflammatory and immunosuppressive qualities of PDN [14], that the taurine in the combined tau + PDN group would then be free to exert its own anti-inflammatory and anti-oxidative properties [8,11,27,28], synergistically reducing the pathology of the *mdx* mouse, as seen previously in adult mice [14]. That a benefit was seen with PDN only, and not with tau + PDN, suggests that the addition of taurine lies in reducing the effect of PDN in this model, and that the substances are competing or inhibiting one another.

It is possible that the adverse effects of a potent steroid counteract any benefit of taurine in the 28 days *mdx* mouse, potentially inhibiting specific pathways benefited by taurine. While no side effects manifested in the current study with methylprednisolone treatment, a worsened dystrophic state has been observed previously in 6 weeks old *mdx* mice following 3 weeks of the daily administration of the anabolic steroid nandrolone decanoate (0.15 mg/100 g body weight) [29]. Ultimately, however, these results suggest that 14 days postnatal taurine supplementation is not effective in the 28 days *mdx* mouse model of DMD.

The 28 days *mdx* mice produced a significantly greater proportion of their maximum force at lower stimulation frequencies (10 and 20 Hz) compared to the WT (Figure 2). These findings are consistent with prior studies that reported EDL muscles from exercised adult *mdx* mice have a more negative mechanical threshold for muscle contraction [14,16,27], resulting in the generation of greater percentages of maximum force at lower frequencies. Interestingly, no difference was observed between the *mdx* and the *mdx* tau, *mdx* PDN, and *mdx* tau + PDN groups, indicating that these treatments were ineffective at ameliorating the altered E-C coupling observed in the *mdx* mouse.

While there was a trend for increased level of fatigue in WT mice at the end of the 180 s fatigue protocol compared to untreated *mdx* mice, there was no significant difference in fatigability across all groups (Figure 3). Here, we did not observe the highly fatigable nature of *mdx* mice that has been reported previously in older animals [30,31], which is likely a reflection of the nature of the fatigue protocol, as well as the age of mice used.

Post tetanic potentiation was observed in all groups during the fatigue recovery period (Figure 3). This has been attributed to increased phosphorylation of the myosin light chains in the contractile apparatus following a sustained period of repeated stimulation. Subsequently, the contractile filaments become more sensitive to Ca^2+^, resulting in a greater amount of cross bridge cycling, which in turn results in a stronger contraction [32,33].

## 5. Conclusions

When taurine was given at 14 days of age for 14 days, and PDN given at 18 days for 10 days, taurine, PDN, and the combined tau + PDN treatment were ineffective in a preclinical model of DMD at attenuating the force loss experienced by *mdx* mice. This is likely because the supplementation occurred in too close a proximity to the onset of severity muscle degeneration, as taurine has been found to be beneficial when supplemented prenatally. Findings further suggest that the benefit of taurine lies in its ability to reduce the initial onset of disease severity, as opposed to attenuating damage or downstream pathways once the pathology has started to progress. Similar to the DMD disease, skeletal muscles of *mdx* mice exhibit myofibre necrosis, varying fibre size, and an increase in centrally located nuclei. Despite the absence of dystrophin in skeletal and cardiac muscles, adult *mdx* mice do not exhibit the progressive characteristics of human DMD as necrosis and regeneration in skeletal muscles peaks around 3–4 weeks of age, and plateaus thereafter. Due to these significant differences in dystrophic pathology, any clinical implications cannot be confidently made until taurine is investigated in a pre-clinical model that has the full spectrum of DMD pathology, such as the mdx^4cv^/mTR^G2^ mouse model [34]. In regard to this, the findings of this study highlight the importance of considering age, life history traits, pathological progression, and most importantly, the time of treatment intervention and analysis when attempting to draw conclusions from disease state animal models. An investigation into the effects of taurine supplemented prenatally and not thereafter would be a valuable addition to this field of research.

## Figures and Tables

**Table 1:** Body mass, tibialis anterior (TA) mass, twitch and tetanic contractile properties of TA muscles from 28 days WT, *mdx*, *mdx* tau, *mdx* PDN, and *mdx* tau + PDN treated mice. L_o_, cross sectional area (CSA), P_t_, TTP and ½RT. n = number of mice. Values are means ± SD; One-way ANOVA with Sidak’s post-hoc analyses between age groups.

-	Body Mass (g)	TA Mass (mg)	L_o_ (mm)	CSA (mm)	P_t_ (mN)	TTP (ms)	½RT (ms)
WT (n = 10)	14 ± 2	20.6 ± 3	10 ± 0.6	3.3 ± 0.4	118 ± 20	26 ± 5	21 ± 5
*mdx* (n = 8)	15 ± 1	23.5 ± 3	11 ± 0.5 *	3.4 ± 0.3	109 ± 33 ^^^	29 ± 3 *	32 ± 10 *
*mdx* tau (n = 6)	13 ± 2	19.3 ± 5	10 ± 0.8	3.0 ± 0.8	91 ± 18 ^^^	27 ± 3	28 ± 10
*mdx* PDN (n = 6)	15 ± 2	25.3 ± 4	11 ± 0.5	3.7 ± 0.4	150 ± 20	29 ± 2	30 ± 5
*mdx* tau + PDN (n = 6)	13 ± 2	19.0 ± 6	10 ± 0.5	3.0 ± 0.8	109 ± 14 ^^^	29 ± 3	32 ± 8

Symbols for significant differences (*p* < 0.05) are: * significantly different from WT group; ^^^ significantly different than *mdx* PDN group.

**Table 2:** 60 Hz fatigue and recovery from 28 days WT, *mdx*, *mdx* tau, *mdx* PDN and *mdx* tau + PDN treated mice. Data expressed as mean percentage force (+/− SD) relative to time zero ± standard error (WT n = 8, *mdx* n = 8, *mdx* tau n = 6, *mdx* PDN n = 6, *mdx* tau PDN n = 6). One-way ANOVA with Sidak’s post-hoc analyses within relevant groups.

-	Fatigue (mN)				Recovery (mN)		
-	0 (s)	60 (s)	120 (s)	180 (s)	240 (s)	300 (s)	360 (s)
WT	1	74 ± 12	71 ± 15	64 ± 9	106 ± 19	108 ±20	107 ± 17
*mdx*	1	87 ± 14	88 ± 19	87 ± 24	112 ± 20	131 ± 40	134 ± 37
*mdx* tau	1	83 ± 7	82 ± 8	79 ± 10	102 ± 12	106 ± 10	108 ± 10
*mdx* PDN	1	80 ± 4	78 ± 5	76 ± 7	98 ± 20	104 ± 18	106 ± 15
*mdx* tau + PDN	1	80 ± 13	77 ± 19	69 ± 26	99 ± 32	108 ± 30	112 ± 29
